# Double Fascicular Nerve Transfer to Musculocutaneous Branches for Restoration of Elbow Flexion in Brachial Plexus Injury

**DOI:** 10.7759/cureus.4517

**Published:** 2019-04-22

**Authors:** Pavlos Texakalidis, Muhibullah S Tora, Jason Lamanna, Jeremy S Wetzel, Nicholas M Boulis

**Affiliations:** 1 Neurosurgery, Emory University Hospital, Atlanta, USA; 2 Neurosurgery, Emory University School of Medicine, Atlanta, USA

**Keywords:** oberlin, elbow flexion, ulnar nerve, median nerve, musculocutaneous

## Abstract

Background

Restriction of elbow flexion significantly limits upper extremity function following brachial plexus injuries. In recent years, the double fascicular nerve transfer procedure utilizing ulnar and median nerve transfer to musculocutaneous branches has shown promising functional outcomes.

Objective

To evaluate restoration of elbow flexion following a double fascicular transfer in patients with brachial plexus injuries and identify predictors of poor outcomes.

Methods

This retrospective review included 10 consecutive patients with brachial plexus injuries involving C5-C6 root avulsions who underwent the double nerve transfer procedure. The mean follow-up was 12 months and the primary outcome was assessment of elbow flexion with the use of the Medical Research Council (MRC) scale.

Results

This procedure achieved elbow flexion of MRC grade M3 or higher in 50% of our cohort. Time interval from injury to surgery showed a statistically significant inverse association with functional recovery (r = -0.73, p = 0.016). Patients who had the surgery within six months of the injury, demonstrated higher MRC grades during the follow-up (p = 0.048). There was no association between elbow flexion recovery and age, body mass index (BMI), gender, hypertension, diabetes or smoking status.

Conclusions

The double fascicular transfer to musculocutaneous may be a safe and effective treatment for restoration of elbow flexion. The procedure is associated with superior functional outcomes when performed within the first six months from the injury.

## Introduction

Brachial plexus injuries can result in significant physical disability, pain and psychological distress [1]. The majority of cases are attributed to motorcycle and motor vehicle traffic accidents [2]. Upper trunk or C5-C6 nerve root injuries can cause loss of elbow flexion, shoulder abduction and external rotation [3]. Despite the fact that hand function can remain intact, restriction of elbow flexion can significantly limit upper extremity usability. Restoration of elbow flexion is commonly given priority over extension, as elbow extension is aided by gravity.

Nerve transfer procedures have been a successful surgical treatment strategy for restoration of elbow flexion. Healthy donor nerves can be transferred onto the damaged musculocutaneous nerve to achieve biceps muscle re-innervation and elbow flexion [4]. The ulnar, medial pectoral, spinal accessory and intercostal nerves can act as potential nerve donors. The Oberlin procedure consists of an ulnar nerve fascicle transfer to the musculocutaneous branch to the biceps and has since been proven to be a safe and effective procedure for restoration of elbow flexion [5,6]. Several studies have shown that the additional transfer of median nerve fascicles to the motor branch to the biceps may be associated with superior outcomes compared to the traditional Oberlin procedure, without an increase in complication rates [4,7]. The aim of this study is to report restoration of elbow flexion following a double fascicular - ulnar and median to musculocutaneous nerve - nerve transfer after brachial plexus injuries and identify predictors of poor outcomes.

## Materials and methods

Study design and patient population

This was a retrospective study of 10 patients with brachial plexus injuries involving the C5-C6 roots who underwent a double fascicular transfer procedure between 2010 and 2017. Medical records were retrieved from the Emory University hospital database after approval of the study protocol by the Institutional Review Board. Experienced abstractors extracted all relevant information including baseline, procedural and outcome data using the electronic medical records database.

All patients lacked elbow flexion prior to the procedure. Clinical examination was performed by the senior author (NMB) both pre- and post-operatively during the long-term follow-up. Electrophysiological studies and computerized tomography myelogram were routinely performed to establish whether lesions were pre- or post-ganglionic and identify the presence of root avulsions.

Data abstraction variables included demographic data (sex, age, body mass index (BMI), hypertension, diabetes mellitus and smoking), mechanism of injury, time interval from injury to surgery and duration of follow-up. All surgeries were performed by NMB.

Surgical technique

**Figure 1:**
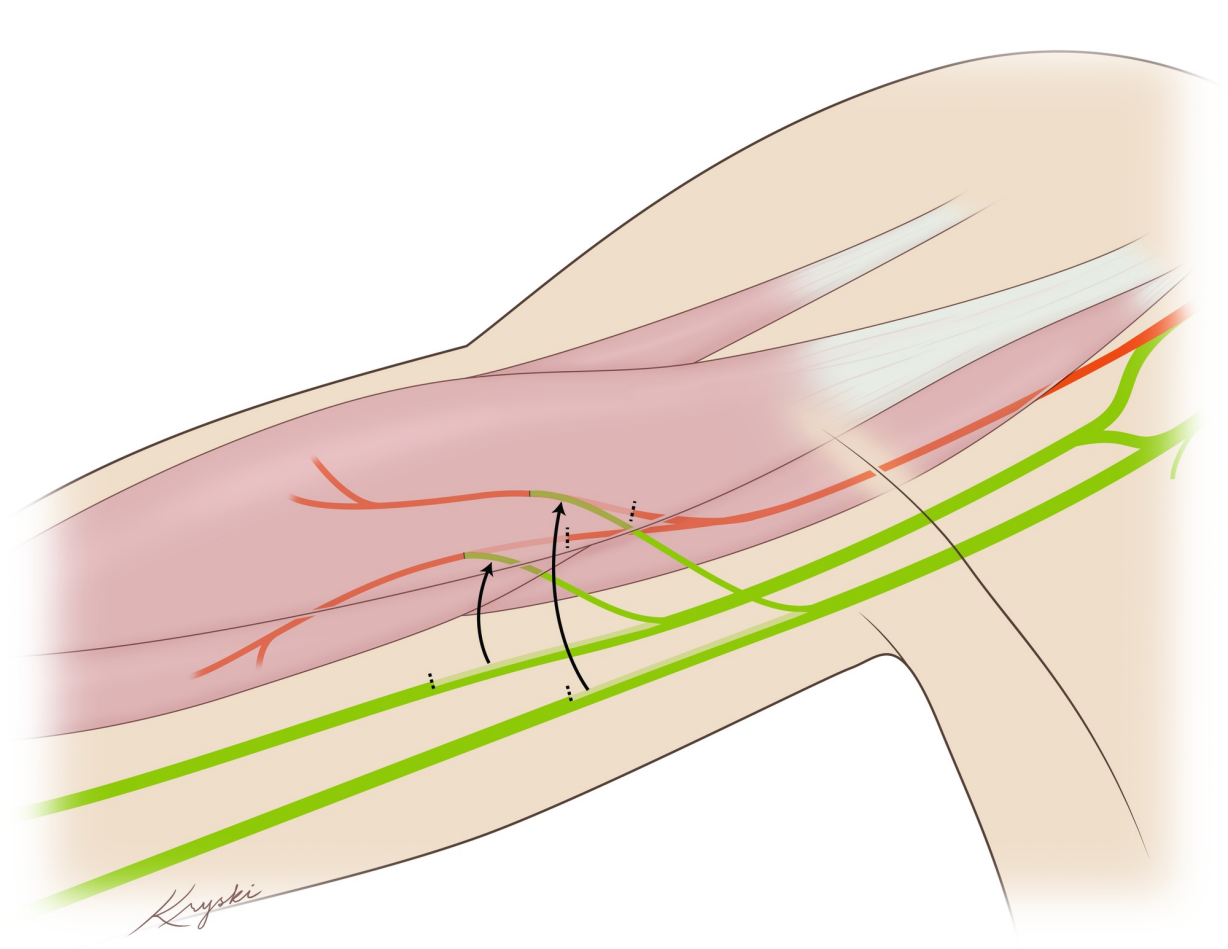
This illustration shows the ulnar and median fascicular transfers to the two branches of the musculocutaneous nerve.

Patients are positioned supine with the right arm abducted and externally rotated. The ulnar and median nerves are identified and dissected circumferentially. Monopolar stimulation reveals muscle contractions in the median and the ulnar nerve. Next, the musculocutaneous nerve is identified and dissected proximally. An internal neurolysis is performed on the musculocutaneous nerve to separate the individual fascicles with branches to the biceps, branches of the brachialis, and the sensory branch under microscopic magnification. Nerve action potentials are performed, confirming conduction in the median and ulnar but not the musculocutaneous nerve. Provided these conditions are met, internal neurolysis of the median and ulnar nerves is performed under the microscope to separate the individual fascicles. One fascicle from each nerve determined by combined motor action potentials (CMAP) to contain the greatest percentage of wrist flexor innervation is cut distally. Nylon 9-0 is used to anastomose the median fascicle to the brachialis and ulnar fascicle to the biceps branches of the musculocutaneous nerve, respectively. Each anastomosis is then secured with Tisseel. Figure 1** **illustrates the double fascicular transfer to musculocutaneous branches.

Definitions and outcome assessment

All patients were evaluated at regular post-operative intervals (mean follow-up: 12 months, range: 4-25). Elbow flexion was assessed by the Medical Research Council (MRC) scale, with scores ranging from 0 (no evidence of contractility) to 5 (full range of motion against gravity with full resistance) [8].

Statistical analysis

Continuous variables were described with the mean ± standard deviation (SD) and compared with the Wilcoxon rank sum rest. Correlation between quantitative variables was assessed with the Spearman’s correlation coefficient test. Categorical variables were described with absolute and relative frequencies. For all tests, p < 0.05 was considered statistically significant. All analyses were performed using STATA software (Version 14.1; Stata Corporation, College Station, TX).

## Results

In total, 10 patients who underwent a double fascicular transfer procedure were identified, of which nine were male (90%) (mean age: 35.6 ± 16, range: 18-56). Patient baseline demographics are presented in Table 1.

**Table 1:** Patient demographic characteristics. BMI: Body mass index.

Age (years)	35.6 ± 16
BMI	27.1 ± 3.9
Male	90% (N = 9/10)
Smoking history	40% (N = 4/10)
Hypertension	60% (N = 6/10)
Diabetes	1% (N = 1/10)

All patients sustained a brachial plexus injury following road traffic accidents (90%) except for one patient who experienced a fall from height. Root involvement levels are outlined in Table 2. Electromyography (EMG) findings showed that nine patients (90%) had sustained root avulsions without axonal continuity to the biceps muscle, of which seven sustained C5-C6 root avulsions, one C5-C7 avulsions, and one patient had a C5 root avulsion. In this cohort, only one patient had no root avulsions where EMG studies showed post-ganglionic C5-C6 involvement.

**Table 2:** Root involvement among patients who underwent double Oberlin nerve transfer.

Root level	N (%)
C5-C6	6 (60%)
C5-C7	3 (30%)
C5-C8	1 (10%)

**Figure 2:**
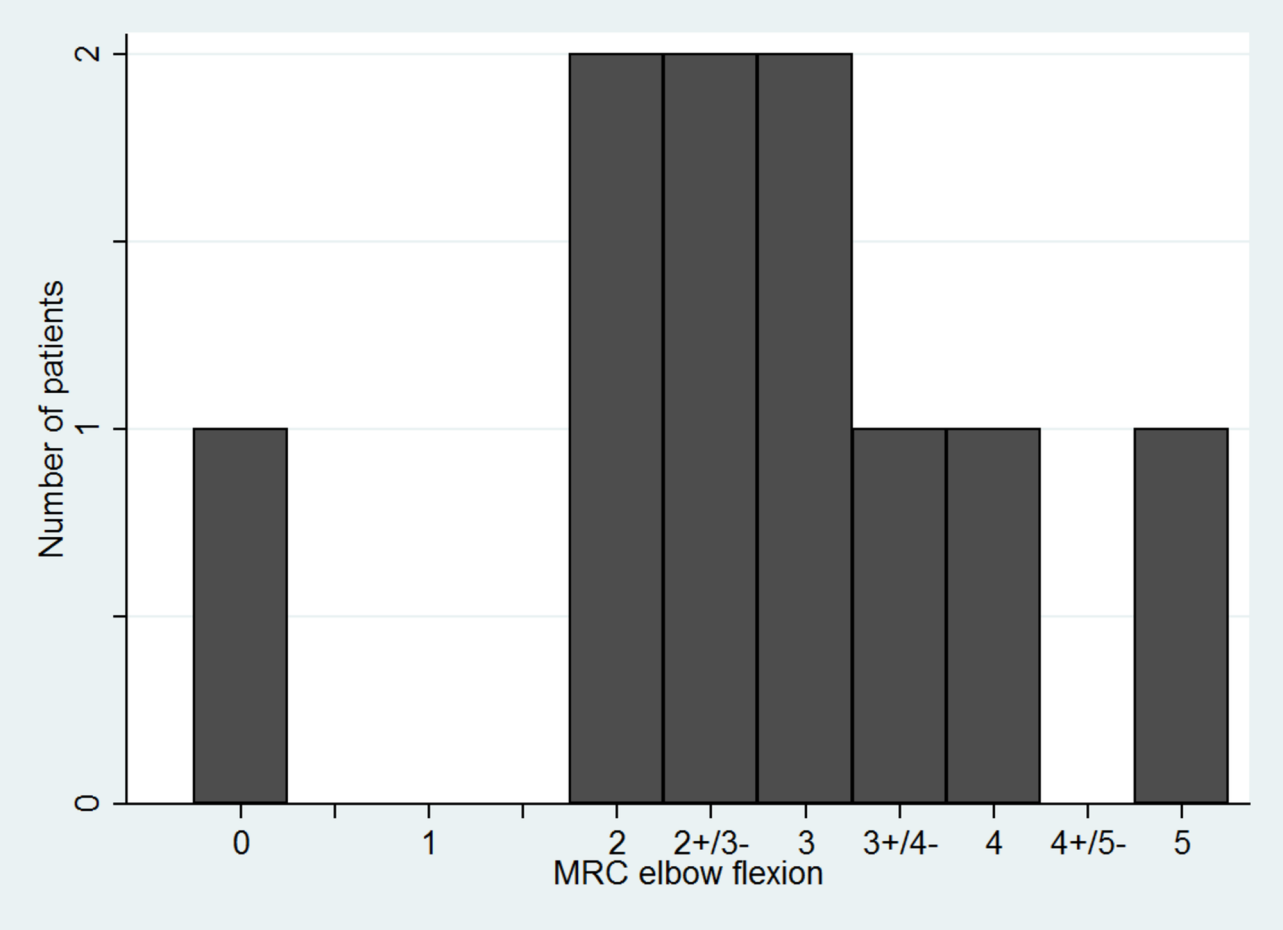
Histogram demonstrating the MRC recovery grade of elbow flexion among our patient cohort. MRC: Medical Research Council.

During the mean follow-up of 12 months (range: 4-25), 50% (N = 5/10) of patients achieved elbow flexion MRC grade M3 or higher which was considered a good outcome. All patients had a pre-operative MRC grade of M0. MRC grades achieved after follow-up in our cohort is shown in Figure 2.

No motor or sensory deficits associated with the ulnar and median nerve were identified during the immediate post-operative and long-term follow-up.

**Figure 3:**
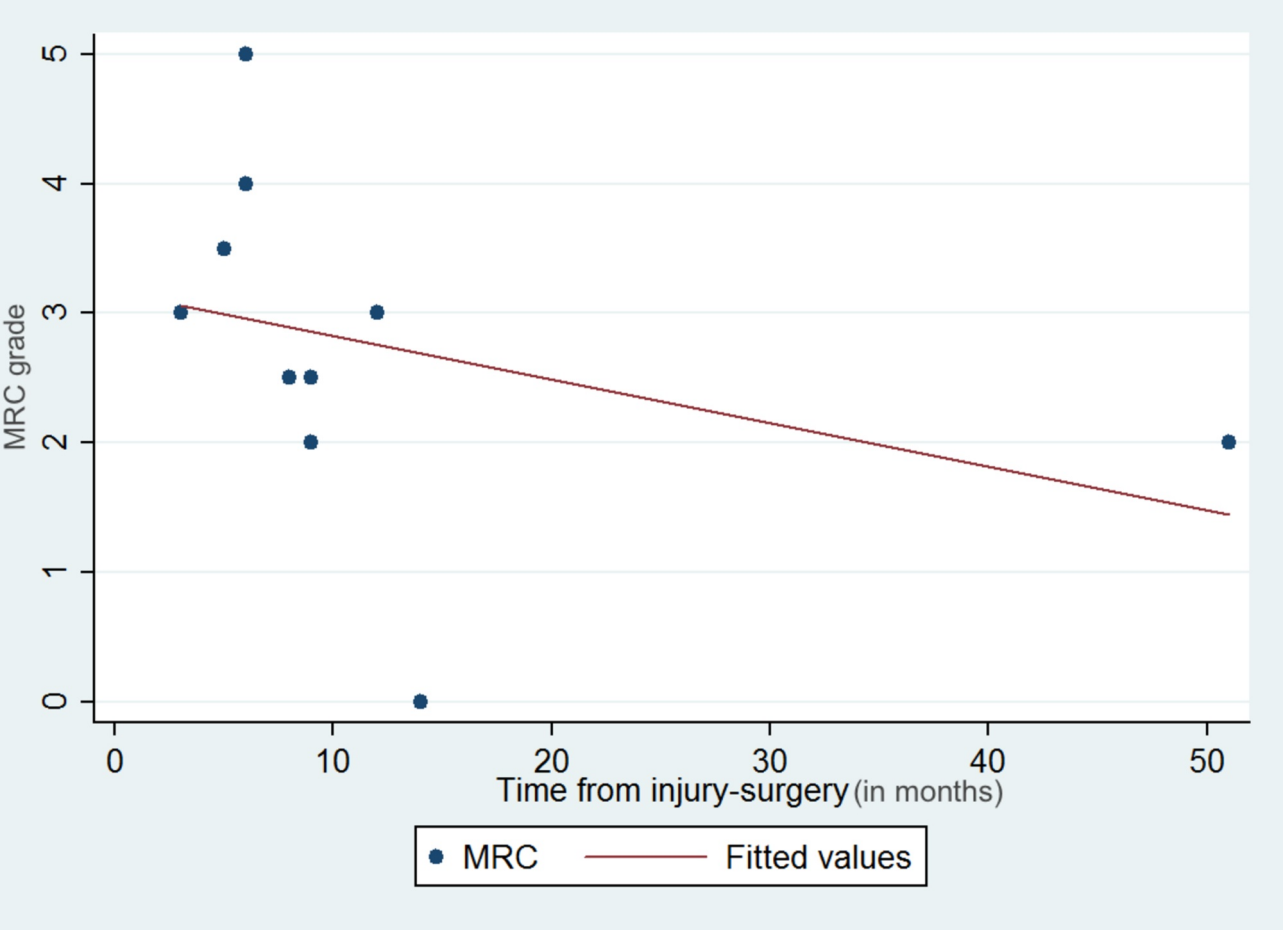
Scatter plot with the best fit line demonstrating the negative linear association between MRC grade recovery and time from injury to surgery. MRC: Medical Research Council.

There was a statistically significant association between MRC elbow flexion grade and time interval from injury to surgery (r = -0.73, p = 0.016) (Figure 3).

**Figure 4:**
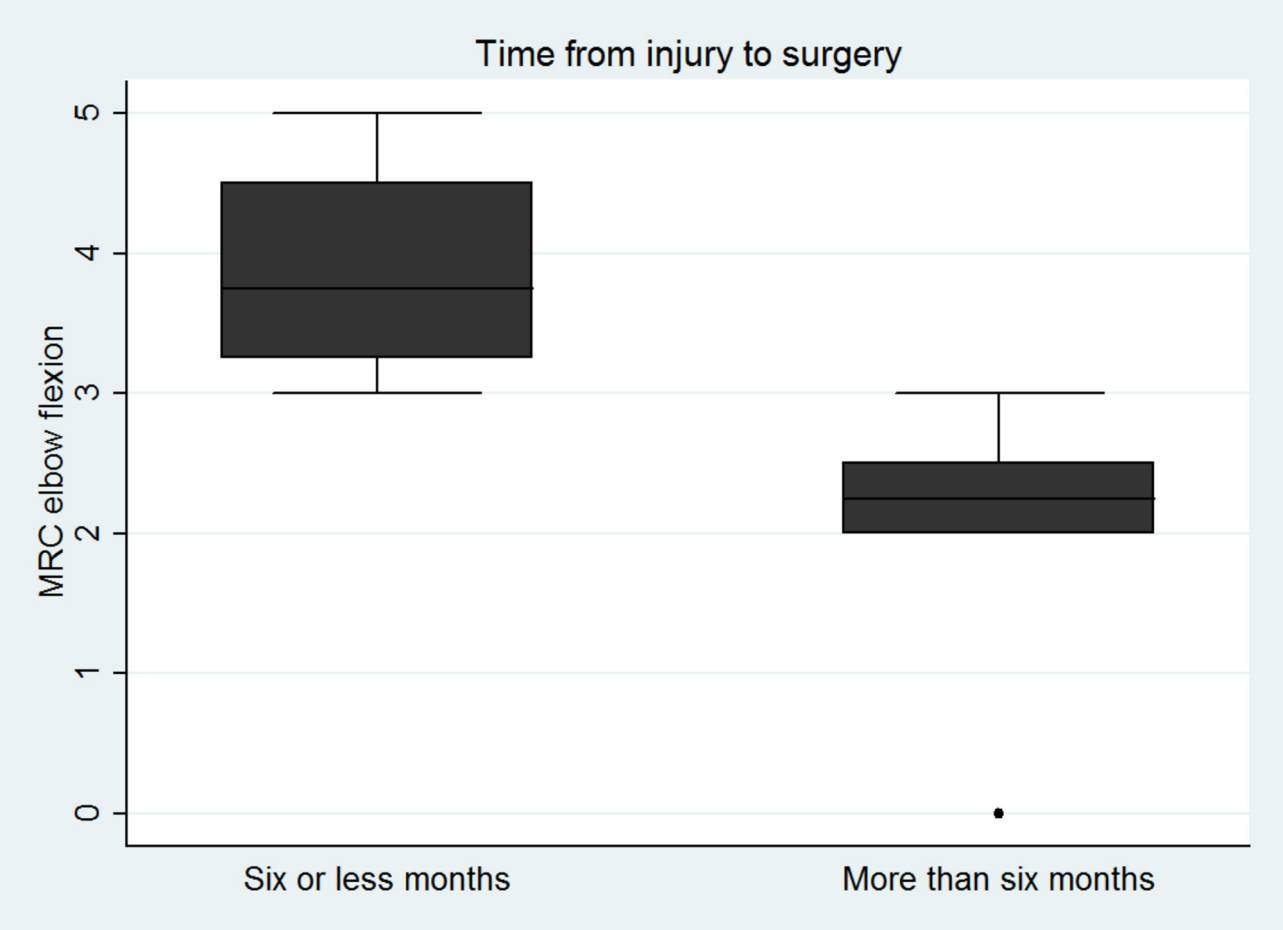
Box plots showing the distribution of MRC elbow flexion grades in patients who had the surgery with less vs. more than six months of their injury. MRC: Medical Research Council.

A separate analysis by categorizing patients in two groups (six or less months vs. more than six months interval between injury and surgery) demonstrated that patients who had the double fascicular transfer within a six or less month time interval from injury to surgery had statistically significant better functional MRC outcomes (p = 0.048) (Figure 4).

No association was demonstrated between MRC grade and patients’ age (r = 0.05, p = 0.89) or BMI (r = -0.04, p = 0.90). Finally, no significant association between MRC elbow flexion and diabetes (p = 0.72), hypertension (p = 0.10), history of smoking (p = 0.74) and male gender (p = 0.21) was identified in our patient cohort.

## Discussion

In this retrospective study, the double ulnar and median fascicle transfer to the musculocutaneous brachialis and biceps branches achieved functional elbow flexion (M3 or higher) in 50% of patients during a mean follow-up of 12 months. There was a statistically significant association between the time interval from injury to surgery and MRC elbow flexion recovery. As previously reported in nerve repair procedures, patients who had surgery within six or less months from their injury, had a higher MRC grade. The presence of diabetes, hypertension, history of smoking and male gender does not seem to affect recovery.

Oberlin’s technique was first described in 1994 and involved transferring ulnar fascicles to the biceps muscle [6]. In the recent years, this procedure has been modified by the addition of median nerve fascicle transfer to the musculocutaneous nerve showing promising results [9,10]. Several studies have suggested that the double transfer may in fact be associated with superior outcomes as compared to the single transfers [11,12]. The study by Mackinnon et al. reported a mean elbow flexion of MRC grade 4+ during the 5.5-month follow-up after a double nerve transfer procedure [12]. Similarly, Liverneaux et al. in a study of 10 patients showed that the double transfer procedure resulted in a mean MRC grade of M4 [11]. In contrast, a prospective study by Martins et al. did not show significant differences between the single and double nerve transfers [13]. Nevertheless, comparative studies are limited in the literature as double transfer is preferentially utilized in most institutions. Future prospective cohorts are warranted to elucidate whether the double transfer is indeed associated with superior functional elbow flexion recovery.

The present study suggested that time interval between injury and surgery can adversely affect recovery of elbow flexion (r = -0.73, p = 0.016). Additionally, this was validated when our cohort was divided into two groups; those who had surgery prior vs. after six months of their injury where we showed that patients who had the early surgery (six months or less) had superior functional outcomes in terms of MRC grade (p = 0.048). Our results are in agreement with previous studies which have reported better functional recovery when the procedure was undertaken within the first six months [5]. Unlike nerve repair procedures, Oberlin transfer involves the use of healthy donor fascicles, thus eliminating the impact of time since injury on the motor neuron. Similarly, distal transfer brings the donor fascicle close to the end plate, and thus should minimize the effect of time since injury on the distal nerve. Given these facts, one would predict that time since injury would affect distal transfers less. In contrast, the motor end plate faces significant changes after the six-month interval and might become irreversible after 12 months [14,15]. This phenomenon, along with ongoing muscle fibrosis, can render the muscle non-responsive to neural stimulation and greatly diminish the potential for re-innervation after nerve transfer procedures, potentially explaining the impact of time since injury on distal transfer [16].

It is worth highlighting that we did not find any association between MRC grade and age following the double nerve transfer procedure (r = 0.05, p = 0.89). Our results are in agreement with previous studies showing that older patients have similar restoration of elbow flexion following a double fascicular transfer [5]. However, results are inconclusive across the literature. Specifically, the study by Liverneaux et al. suggested that older patients may have a decreased potential for restoration of elbow flexion following an Oberlin transfer [11]. Future studies with a larger patient sample are needed to identify if increased age is a predictor of poor functional outcome. Lastly, the present study did not show any association between elbow flexion and patient demographics including BMI, diabetes, hypertension, smoking and gender which is line with studies examining nerve transfer procedures [17,18].

Limitations

Several limitations should be noted for this study. First, our study was retrospective and thus limited by its non-randomized nature. Second, despite the standardized follow-up schedule at our institution, its duration was not similar for all patients due to some patients missing appointments, which might have affected the outcomes. Third, this study is limited by its low patient sample, single surgeon experience and lack of matched controls.

## Conclusions

The current study suggests that the double ulnar and median nerve transfer to the musculocutaneous may be a safe and effective approach for elbow flexion restoration following C5-C6 root avulsions. Also, it points out that functional outcomes are adversely affected by the increase in the time interval from injury to surgery; the double fascicular transfer within the first six months is suggested by this study. No association between MRC grades and patient demographic characteristics was identified.
